# Virucidal efficacy of a UV-C disinfection system for semi-critical medical devices according to EN 14476

**DOI:** 10.3205/dgkh000622

**Published:** 2026-02-06

**Authors:** Stefan Alexander Rudhart, Klaus Wolfgang Georg Eichhorn, Marvin Rausch, Alexander Maas, Maximilian Knof, Steffen Engelhart, Nico T. Mutters, Stephan Hoch, Sebastian Strieth

**Affiliations:** 1 Department of Otorhinolaryngology, University Medical Center Bonn (UKB), 53127 Bonn, Germany; 2 Institute for Hygiene and Public Health, University Medical Center Bonn (UKB), 53127 Bonn, Germany; 3 Department of Otolaryngology, Head and Neck Surgery, University Hospital Marburg, Philipps-Universität Marburg, Baldingerstrasse, 35043 Marburg, Germany; 4 Department of Hearing Disorders, Tinnitus, Vertigo, and Cochlear Implants (HTS), MEDIAN Kaiserberg-Klinik Bad Nauheim, 61231 Bad Nauheim, Germany

**Keywords:** disinfection, EU-Norm, ultraviolet radiation, virucidal efficacy, endoscope reprocessing, semi-critical medical devices

## Abstract

**Purpose:**

Semi-critical medical devices, such as flexible endoscopes in otorhinolaryngology (ORL), require high-level disinfection due to their contact with mucous membranes. UV-C systems offer a rapid, non-toxic alternative to traditional reprocessing. This study evaluates the virucidal efficacy of the D60 UV-C disinfection system according to EN 14476 standards.

**Materials and methods:**

Standardized tests were performed using frosted glass and thermoplastic elastomer (TPE) carriers inoculated with four indicator viruses: murine norovirus (MNV), poliovirus type 1, adenovirus type 5, and simian virus 777 (SV40). After drying, carriers underwent a 60 second UV-C cycle in the D60 system. Viral recovery and titration followed EN 14476 protocols, with infectivity measured by TCID_50_ assay.

**Results:**

All viruses showed significant reductions following UV-C treatment. Residual titers fell below the detection limit (3.2 lg TCID_50_/mL), corresponding to >4.0 lg reduction across all tested surfaces and virus types. Adenovirus showed the highest reduction (>4.5 lg).

**Conclusion:**

The D60 system achieved broad-spectrum virucidal efficacy within one minute, meeting EN 14476 requirements. These findings support its use for safe, rapid reprocessing of semi-critical devices in high-turnover clinical settings, with potential to improve infection control and workflow efficiency.

## Zusammenfassung

**Zielsetzung:**

Semikritische Medizinprodukte wie flexible Endoskope ohne Arbeitskanal in der Hals-, Nasen- und Ohrenheilkunde (HNO) erfordern aufgrund ihres Kontakts mit Schleimhäuten eine high-level Desinfektion. UV-C-Systeme bieten eine Alternative zur herkömmlichen Aufbereitung. Diese Studie bewertet die viruzide Wirksamkeit des D60 UV-C-Desinfektionssystems gemäß den Anforderungen der EU Norm EN 14476.

**Material und Methoden:**

Die standardisierten Tests erfolgten auf mattierten Glas- und thermoplastischen Elastomer-(TPE)-Trägern, die mit vier Indikatorviren – murines Norovirus (MNV), Poliovirus Typ 1, Adenovirus Typ 5 und Simian Virus 777 (SV40) – beimpft wurden. Nach dem Trocknen wurden die Träger einem 60-sekündigen UV-C-Desinfektionszyklus im D60-System unterzogen. Die Auswertung erfolgten nach den EN-14476-Protokollen, und die Restviruzidie wurde mittels TCID_50_-Assay bestimmt.

**Ergebnisse:**

Alle getesteten Viren zeigten nach der UV-C-Behandlung signifikante Reduktionen im Virustiter. Die Resttiter lagen unter der Nachweisgrenze (3,2 lg TCID_50_/mL), was einer Reduktion von >4,0 lg auf allen geprüften Oberflächen und für alle Virustypen entspricht. Das Adenovirus zeigte die stärkste Reduktion (>4,5 lg).

**Schlussfolgerung:**

Das D60-System erreichte innerhalb einer Minute eine breit wirksame viruzide Aktivität und erfüllt damit die EU-Anforderungen der EN 14476. Die Ergebnisse untermauern den Einsatz des Systems für eine sichere, effiziente Aufbereitung semikritischer Medizinprodukte in stark frequentierten klinischen Bereichen.

## Introduction

A broad variety of reusable medical devices (MD) requires high-level disinfection prior to reuse. According to the Spaulding classification [1], these so-called semi-critical MD come into contact with mucous membranes or non-intact skin but do not penetrate sterile body areas. Rigid and flexible endoscopes used in otorhinolaryngology (ORL) represent a typical example. These instruments are frequently used for examination of the upper aerodigestive tract, a region particularly susceptible to colonization by a wide array of pathogenic and potentially carcinogenic viruses, including human papillomavirus (HPV), Epstein-Barr virus (EBV), and various coronaviruses [2], [3], [4], [5]. In contrast to non-critical instruments, which only contact intact skin, semi-critical MD present a higher risk of cross-contamination, especially in high-turnover outpatient settings. Reliable, rapid, and user-independent disinfection methods are thus essential in ensuring patient safety. While numerous chemical and thermal methods exist for device reprocessing, these are often costly, time-consuming, or operator-dependent, making their consistent implementation in daily clinical routines challenging.

Ultraviolet (UV) light has long been used for surface disinfection, with its effectiveness against bacteria and certain viruses well documented [6], [7], [8]. In recent years, novel UV-based systems have shown promising as an alternative method for the high-level disinfection of MD, offering advantages such as non-toxicity, no residue formation, efficacy against biofilms and short cycle times [9], [10], [11]. Previous investigations into the bactericidal efficacy of the UV-C–based D25 system (UV Smart Technologies B.V., Rijswijk, Netherlands) demonstrated substantial microbial load reductions, achieving complete elimination of bacterial contaminants in both standardized test specimen and clinical endoscope reprocessing scenarios [11], [12]. Initial virucidal evaluations using MS2 bacteriophages as surrogate markers for non-enveloped viruses indicated promising inactivation profiles and laid the groundwork for subsequent studies [13]. In the current investigation, we evaluated the D60 system—a further development of the D25 platform specifically optimized for flexible otorhinolaryngological endoscopes and transesophageal echocardiography (TEE) ultrasound probes—which operates on the same UV-C photoinactivation principle. Although only phage-based surrogate data have been reported for the D25 device, the D60 system has already been shown to possess potent bactericidal efficacy under comparable test conditions [12], [13].

However, to meet the stringent requirements set forth by European regulatory frameworks—specifically the Biocidal Products Regulation (BPR, Regulation (EU) No. 528/2012), disinfection systems must demonstrate efficacy according to standardized test protocols [14]. For virucidal efficacy, the most relevant guideline is EN 14476, which defines a quantitative suspension test under simulated practical conditions. In this context, a product must achieve at least a 4 lg (≥ 99.99 %) reduction in infectivity of defined indicator viruses, which serve as representative models for broader viral inactivation [15].

In order to be classified as “virucidal” according to EN 14476, a disinfection system must demonstrate reliable inactivation of poliovirus type 1, a highly resistant non-enveloped enterovirus; adenovirus type 5, a moderately resistant non-enveloped DNA virus; and murine norovirus (MNV), a stable RNA virus commonly used as a surrogate for human norovirus. Successful inactivation of all three viruses provides confirmation of broad-spectrum virucidal activity, encompassing both enveloped and non-enveloped pathogens. In addition to these mandatory test organisms, Simian Virus 777 (SV40) was included in this study as a supplementary indicator virus. SV40 is a non-enveloped, double-stranded DNA virus from the Polyomaviridae family, known for its pronounced environmental stability and resistance to standard disinfection procedures. Due to its structural and physicochemical similarities to human polyomaviruses and adenoviruses, SV40 is occasionally employed in virucidal efficacy studies as an additional benchmark for the inactivation of resilient DNA viruses [15], [16], [17].

In the present study, we evaluated the virucidal performance of the D60 UV-C disinfection system in strict accordance with these European testing standards. By directly employing the EN 14476 indicator viruses, we provide an accurate, regulatory-compliant assessment of the D60 system’s capacity to fulfill the criteria for high-level virucidal disinfection. This investigation represents a critical step toward validating UV-based reprocessing technologies for semi-critical devices in ORL and related high-risk clinical settings. 

## Materials and methods

### Experimental conditions

**Figure 1:**
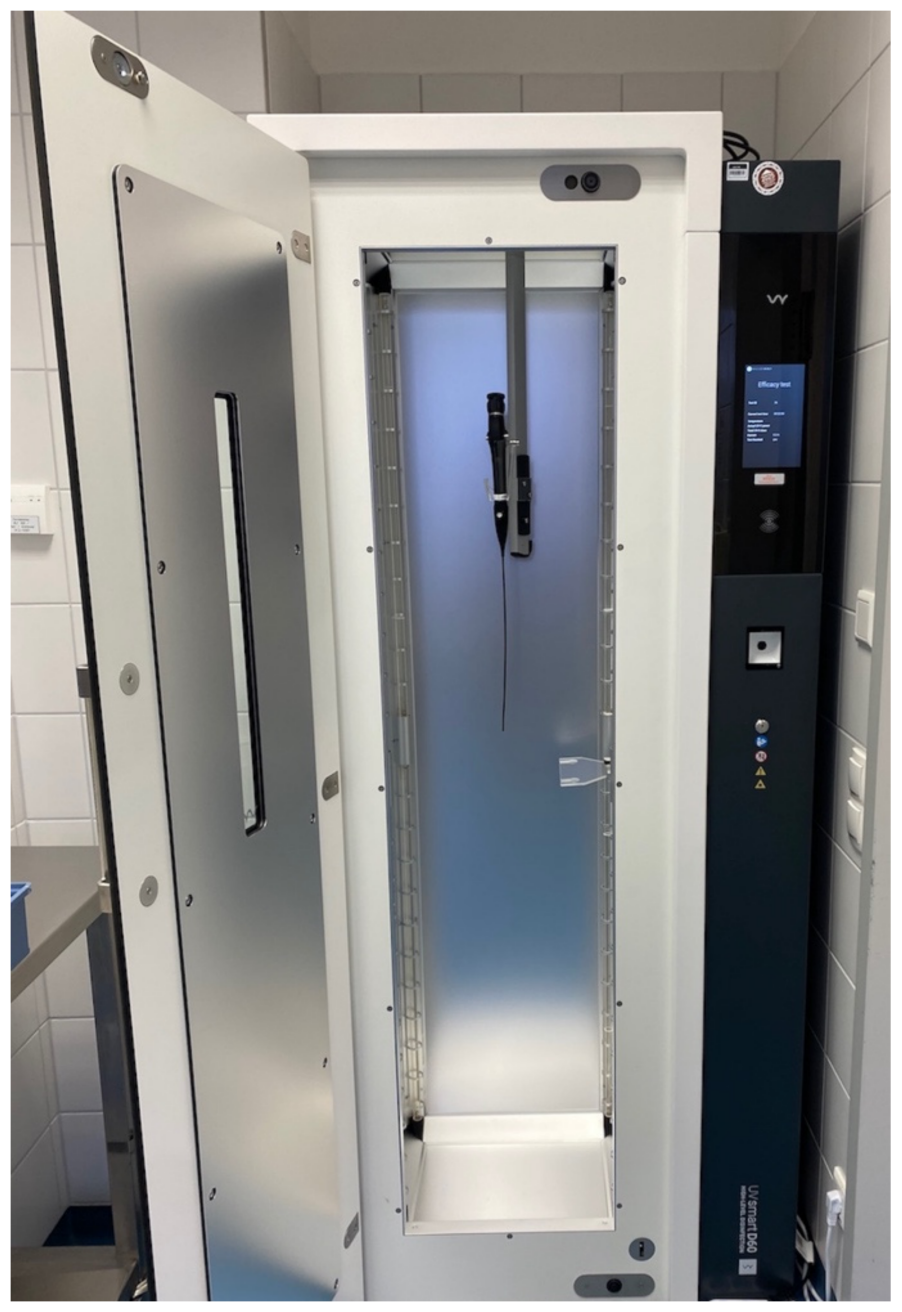
D60 UV disinfection system (UV Smart Technologies B.V., Rijswijk, Netherlands) with a thermolabile flexible otorhinolaryngological endoscope inside, as it is used in clinical routine.

The virucidal efficacy of the D60 UV-C disinfection system (UV Smart Technologies B.V., Rijswijk, Netherlands) (Figure 1 (Fig. 1)) was evaluated under standardized experimental conditions. A 60 seconds standard cycle, as preset on the device, was used for all experiments. Tests were conducted at room temperature (18 °C–25 °C) in the presence of bovine serum albumin (BSA) at a final concentration of 0.3 g/L (0.03 %), to simulate “clean” interfering conditions according to EN 14476.

### Test surfaces and carriers

**Figure 2:**
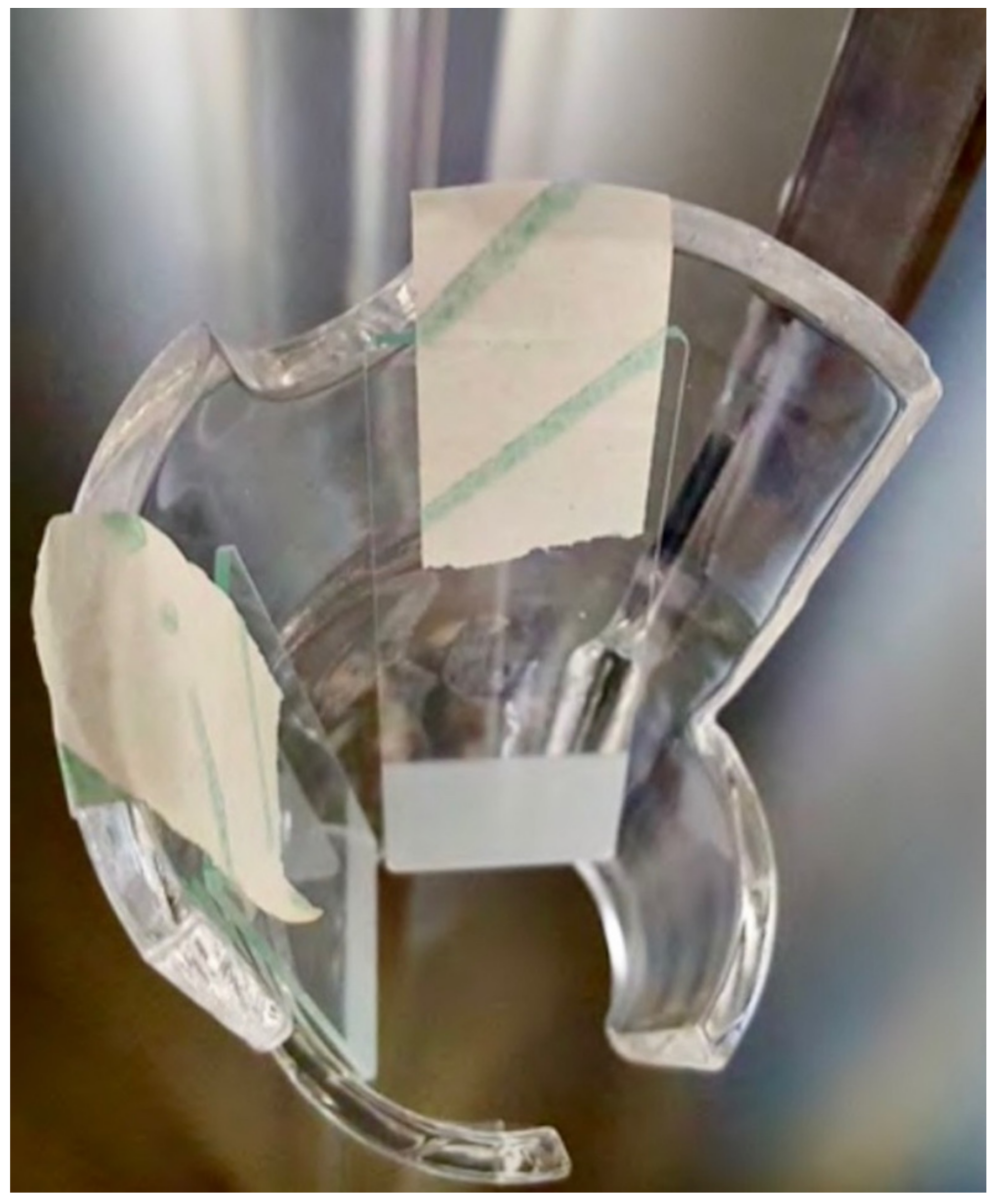
Frosted glass slides (15 mm×60 mm×1 mm, one side sandblasted), arranged in the D60 disinfection system hanger

**Figure 3:**
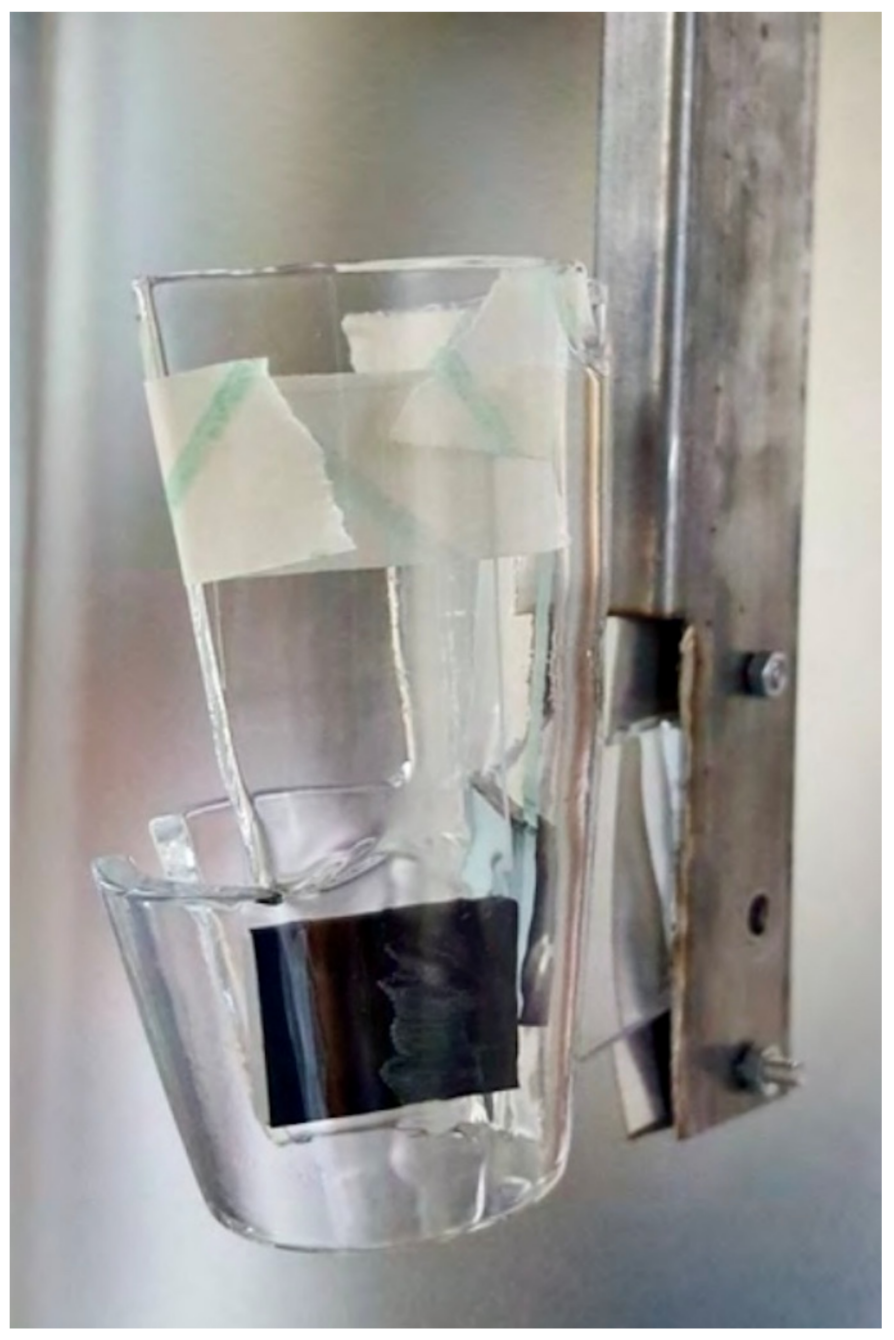
Thermoplastic elastomer (TPE 13(2)) samples, arranged in the D60 disinfection system hanger

Two types of carriers were employed: frosted glass slides for representation of hard non-porous surfaces (15 mm×60 mm×1 mm, one side sandblasted (Figure 2 (Fig. 2)), supplied by Eurofins (Luxemburg, Luxemburg, Bioanalytics), and thermoplastic elastomer (TPE 13(2)) samples for representation of the endoscope surface (Figure 3 (Fig. 3)). Prior to inoculation, all carriers were handled under a laminar-flow cabinet to ensure sterility.

### Viruses and host cells

The following indicator viruses were tested in accordance with EN 14476 requirements: Adenovirus Type 5 (ATCC VR-5), Poliovirus Type 1 LSc-2ab (RVB-1260), Murine Norovirus strain S99 (RVB-651), and Simian Virus 777 (SV40). Each viral stock exhibited an initial titer of ≥10^8^ TCID_50_/mL, ensuring the capacity to detect a ≥4 lg reduction. Host cell lines used for virus titration were HeLa cells (ATCC CCL-2) for Adenovirus, CV-1 monkey kidney fibroblasts for Poliovirus and Simian Virus, and RAW 264.7 murine macrophages (ATCC TIB-71) for Murine Norovirus. All cell cultures were maintained and assayed at 37°C ± °C with 5% CO_2_.

### Inoculation and drying

Each carrier was inoculated with 10 µL of the virus suspension, applied to the sandblasted side at one end while avoiding the edges. Inoculated carriers were left to dry at room temperature under laminar airflow for up to one hour or until visibly dry.

### UV-C treatment and virus recovery

Immediately after drying, two carriers per virus–surface combination were mounted on the device’s holder with the inoculated side facing the UV-C light source. Following the standard one-minute UV cycle, each carrier was transferred into a sterile tube containing 5 mL of ice-cold culture medium. Viruses were eluted by pipetting up and down across the surface for 60 seconds.

### Titration and TCID_50_ determination

Eluates underwent eight tenfold serial dilutions in ice-cold maintenance medium. Each dilution was inoculated sixfold (0.1 mL per well) onto confluent monolayers (>90%) in 96-well microplates without additional medium. After a one-hour adsorption period at 37°C ±1°C, 0.1 mL of maintenance medium was added to each well. Wells in the outermost rows received only medium as negative controls. Plates were incubated for the virus-specific period, then examined by inverted microscopy for cytopathic effect (CPE). Viral titers (TCID_50_/mL) were calculated using the Spearman-Kärber method.

### Drying controls

To assess virus stability during drying, the same inoculation and drying protocol was applied to two additional carriers per virus–surface pair, which were not exposed to UV-C. One set was sampled immediately after visible drying (time 0), and the second set after one minute (maximum contact time). Virus recovery and titration were performed as described above, and the mean titer served as the drying control (a).

### Validity criteria and data interpretation

An assay was considered valid if initial virus stocks had titers of at least 10^8^ TCID_50_/mL, allowing for detection of a ≥4 lg reduction. The lg reduction I was calculated as the difference between the drying control titer at maximum contact time (a) and the residual virus titer after UV treatment (b):

*R=a - b*

A reduction of ≥4 lg under the defined conditions was the threshold for virucidal activity. If the Sponsor requested additional conditions (e.g., modified temperature, contact time, interfering substance, or extra viral strains), results were reported as “causes” or “does not cause” a ≥4 lg reduction under those specified parameters.

### Ethic

The study was reviewed by the Ethics Committee of the University Hospital Bonn under reference number 2025-85-BO.

## Results

**Figure 4:**
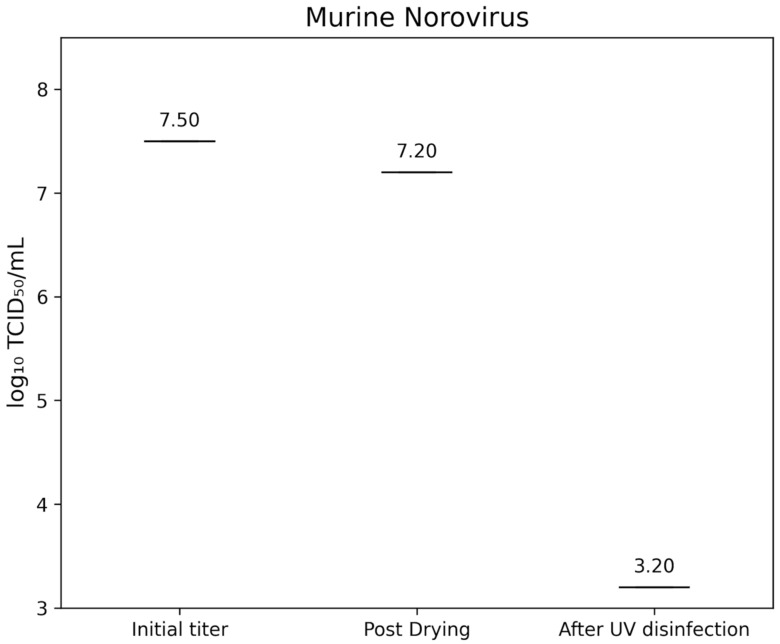
lg TCID_50_/mL titers of Murine Norovirus (MNV) on test surfaces before drying (initial titer), after drying, and following UV-C disinfection

The initial infectivity titer of murine norovirus strain S99 (RVB-651) was 7.50±0.00 lg TCID_50_/mL. After a 60-second drying period on both frosted glass and TPE carriers, the titer decreased to 7.20±0.00 lg TCID_50_/mL. Following a 60-second exposure in the D60 UV-C system, no residual infectivity was detected above the assay limit of 3.2 lg TCID_50_/mL, corresponding to a reduction of >4.0 lg (Figure 4 (Fig. 4)).

**Figure 5:**
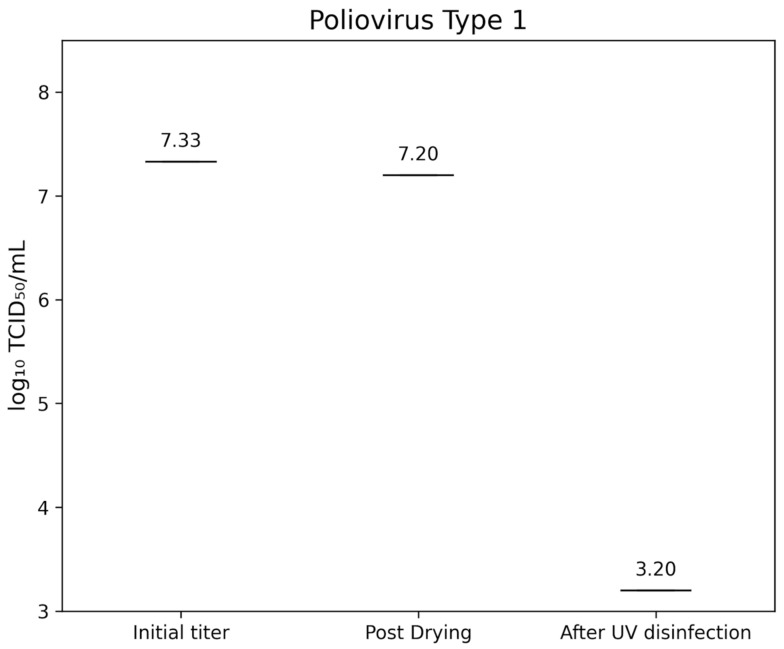
lg TCID_50_/mL titers of Poliovirus Type 1 on test surfaces at initial inoculation, post-drying, and after UV-C treatment

Poliovirus Type 1 LSc-2ab exhibited an initial titer of 7.33±0.346 lg TCID_50_/mL, which decreased to 7.20±0.00 lg TCID_50_/mL after drying. UV-C treatment reduced the residual titer below the detection threshold of 3.2 lg TCID_50_/mL, indicating a >4.0 lg reduction (Figure 5 (Fig. 5)). 

**Figure 6:**
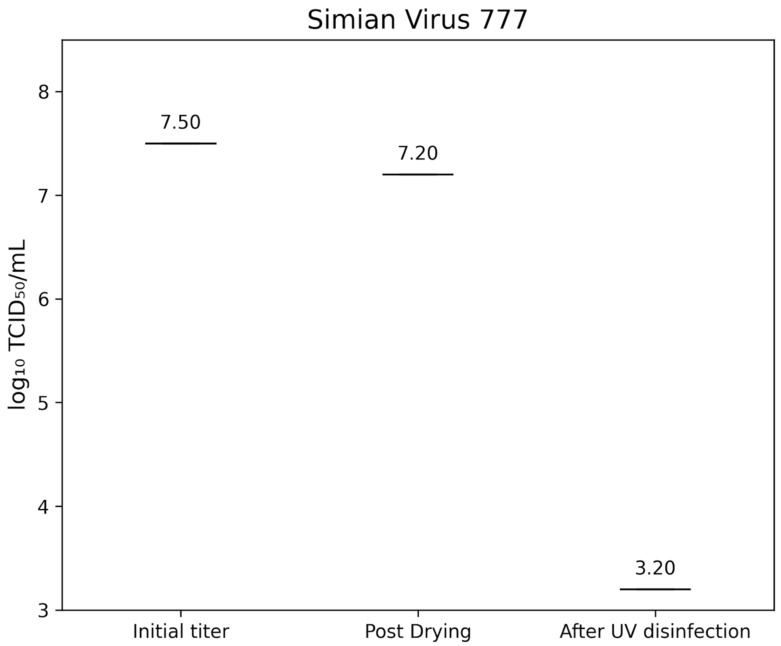
Lg TCID_50_/mL titers of Simian virus strain 777 on test surfaces at initial inoculation, post-drying, and after UV-C treatment

Simian virus strain 777 (SV40) showed an initial titer of 7.50±0.00 lg TCID_50_/mL, decreasing to 7.20±0.00 lg TCID_50_/mL after drying. Post-treatment titers were below 3.2 lg TCID_50_/mL, corresponding to a >4.0 lg reduction (Figure 6 (Fig. 6)).

**Figure 7:**
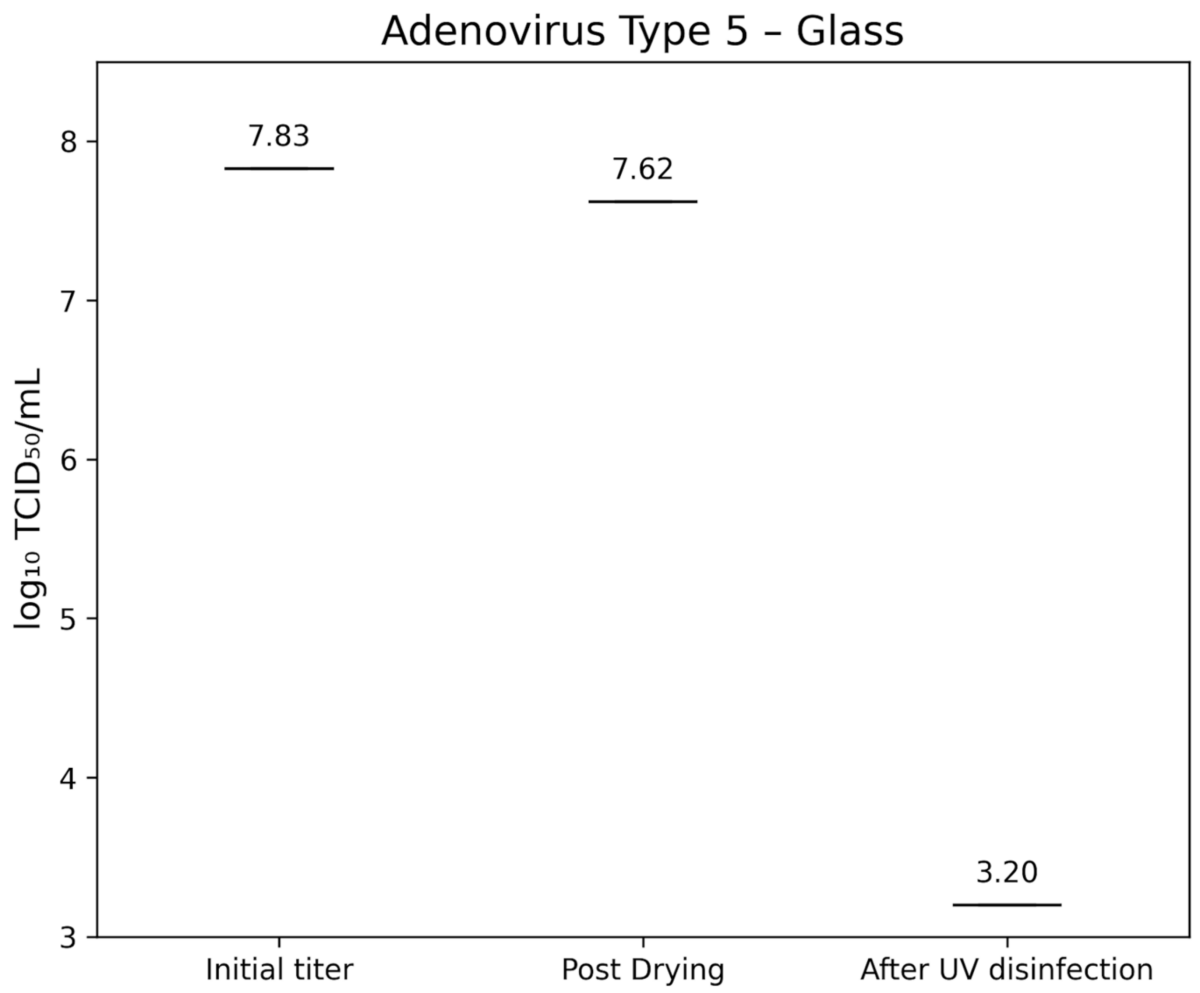
lg TCID_50_/mL titers of Adenovirus Type 5 on glass test surface at initial inoculation, post-drying, and after UV-C treatment

**Figure 8:**
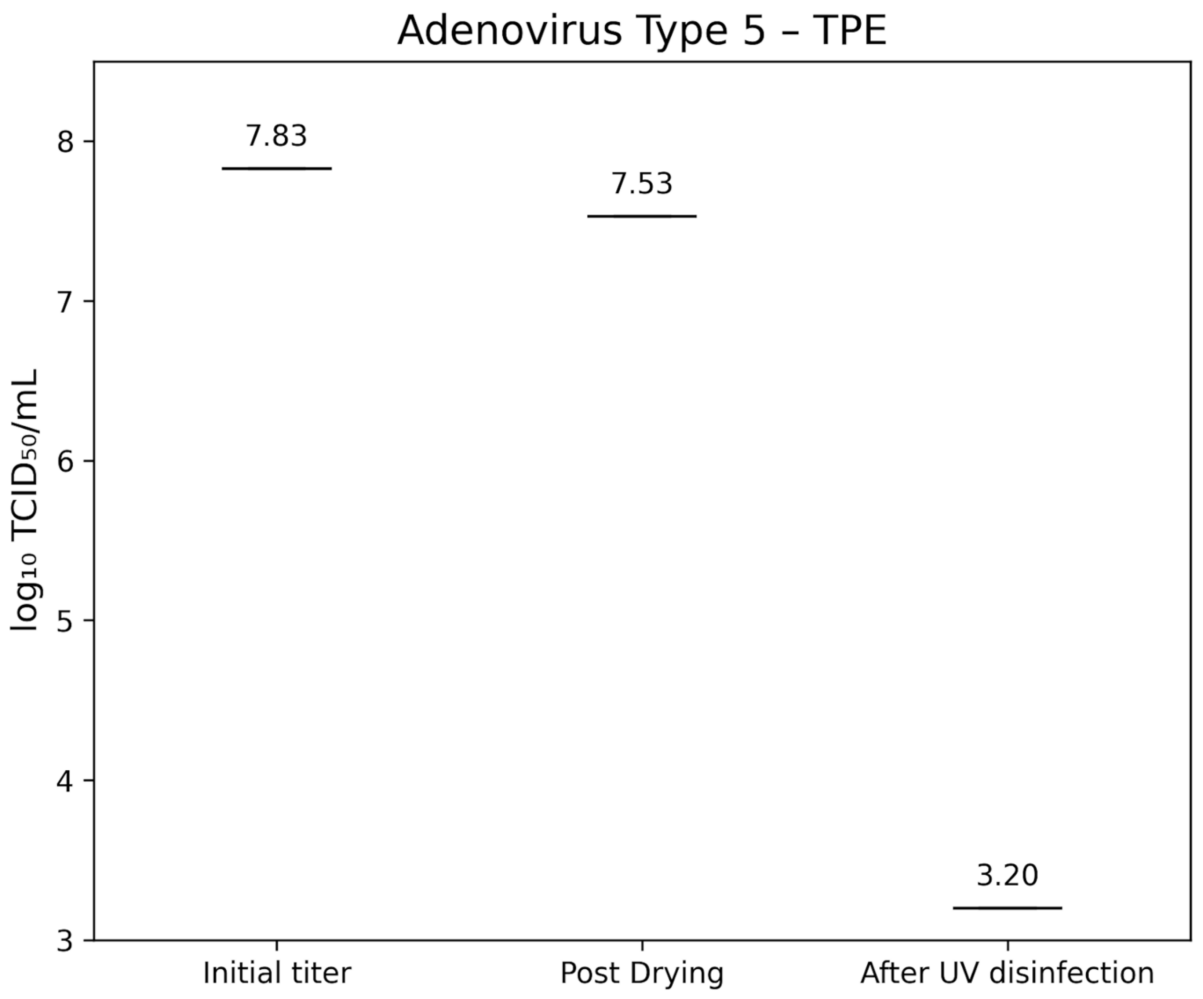
lg TCID_50_/mL titers of Adenovirus Type 5 on TPE-test surface at initial inoculation, post-drying, and after UV-C treatment

Adenovirus Type 5 (ATCC VR-5) had an initial titer of 7.83±0.40 lg TCID_50_/mL. After drying, titers were 7.62±0.30 lg TCID_50_/mL on glass (Figure 7 (Fig. 7)) and 7.53±0.400 lg TCID_50_/mL on TPE (Figure 8 (Fig. 8)). Following UV-C exposure, residual infectivity on both surfaces fell below 3.2 lg TCID_50_/mL, corresponding to a reduction of >4.2±0.224 lg.

## Discussion

The D60 UV-C disinfection system achieved greater than a 4 lg reduction of murine norovirus (MNV, strain S99), poliovirus type 1 LSc-2ab, simian virus 777 (SV40), and adenovirus type 5 under a 60 second cycle on both glass and TPE carriers. These results align with, and in several cases exceed, inactivation profiles reported for comparable UV-C or UVGI systems in the literature. 

Murine norovirus serves as a widely accepted surrogate for human norovirus due to its similar non-enveloped RNA structure and environmental persistence. In our experiments, MNV titers fell from 7.20 lg TCID_50_/mL after drying to below 3.2 lg TCID_50_/mL following one minute of D60 UV-C treatment, indicating a reduction >4.0 lg. Previous surface studies using UVGI reported that single-stranded RNA viruses require relatively low fluences for inactivation: the dose for 90% reduction ranged from 1.32 to 3.20 mJ/cm², with approximately double that fluence for 99% inactivation [18]. Although specific MNV data are less common, Oguma et al. [19] demonstrated with TiO_2_-augmented UV that MNV achieved >4 lg reductions under surface conditions at moderate doses, underscoring UV’s efficacy against noroviruses. The rapid inactivation observed here suggests that the D60 system delivers a UV-C fluence comparable to- or exceeding the ~5–10 mJ/cm² required for 4 lg MNV reduction in other UVGI configurations [20]. Poliovirus type 1 is one of the most UV-resistant non-enveloped RNA viruses commonly used in virucidal testing. In our study, PV-1 titers dropped from 7.20 lg TCID_50_/mL after drying to below 3.2 lg TCID_50_/mL post-UV exposure (>4.0 lg reduction). Former studies reported that feline calicivirus and adenovirus type 40 required UV doses of approximately 50 mJ/cm² for 3 lg and roughly 80–100 mJ/cm² for 4 lg reductions, with poliovirus inactivation kinetics following similar curves [21]. In water and aerosol studies, poliovirus 1 achieved 4 lg reductions at fluences between 20 and 40 mJ/cm² [18], [19], [20], [21]. The D60’s one-minute cycle, therefore, appears to deliver an effective fluence in line with these established thresholds, confirming its suitability for high-level disinfection against robust enteric viruses.

Double-stranded DNA adenoviruses are notably more UV-resistant than single-stranded RNA viruses, often requiring higher fluences for equivalent lg reductions. The D60 system reduced adenovirus type 5 from 7.83 lg to below 3.2 lg TCID_50_/mL (>4.5 lg reduction). Published data indicate that adenovirus type 2 demands ~80 mJ/cm² for 4 lg inactivation under low-pressure UV [22]. Enhanced inactivation using polychromatic UV sources achieved >4 lg reductions at doses as low as 25 mJ/cm², while pulsed UV modalities reported 4 lg declines in the range of 20–40 mJ/cm² [23], [24]. These comparisons suggest that the D60 fluence is at least on par with, and likely superior to, many conventional UVGI systems, supporting its capacity to neutralize DNA viruses effectively within short contact times.

Simian Virus 777 (SV40), another double-stranded DNA virus, is less frequently studied in UV-C contexts but is assumed to share resistance profiles with human adenoviruses. Simian Virus 777 (SV40) exhibits notable environmental persistence and a high level of resistance to conventional disinfection methods [16], [17]. Our SV40 titers decreased by >4.0 lg within one minute of UV-C exposure. Limited SV40 UV studies imply that similar fluences (50–100 mJ/cm²) are necessary for multi-lg reductions, paralleling adenovirus responses [25]. The D60’s demonstrated performance thus aligns with expectations for dsDNA viruses, further validating its broad-spectrum virucidal claims.

Virus inactivation by UV-C principally involves nucleic acid damage, particularly pyrimidine dimer formation in DNA or RNA strands, leading to replication arrest. The differences in UV susceptibility between ssRNA, dsRNA, and dsDNA viruses are well documented: ssRNA viruses (e.g., MNV) are more readily inactivated than dsDNA viruses (e.g., adenovirus, SV40) because of their simpler genome structure and lack of repair mechanisms [20], [22]. Our data reflect these mechanistic trends: MNV and poliovirus were fully inactivated at the assay limit within one minute, whereas adenovirus and SV40 required slightly longer or higher fluence to reach the same endpoint—yet still within the preset cycle time of the D60.

Although our evaluation focused on surface decontamination, it is informative to compare with water and aerosol UV inactivation literature. Poliovirus and adenovirus achieve 4 lg inactivation in water at doses of 20–60 mJ/cm², and aerosolized viruses require 2–5 mJ/cm² for 90% reductions [20]. The D60’s performance on solid carriers thus appears at least equivalent to, if not more efficient than, many fluid or airborne applications, underscoring its utility across disparate disinfection contexts.

Meeting the EN 14476 criterion of ≥4 lg reduction for poliovirus, adenovirus, and murine norovirus is mandatory for “virucidal” claims in the EU [21]. The D60 system’s ability to accomplish this in a one-minute cycle on both glass and TPE surfaces demonstrates its compliance with the Biocidal Products Regulation (EU No. 528/2012) and positions it favorably for certification under EN 14476 [21]. These results also suggest suitability for rapid, high-turnover clinical environments such as ORL outpatient clinics, where flexible endoscopes and TEE probes require efficient reprocessing.

### Limitations and future directions

While these findings are robust, the study did not directly measure UV-C fluence delivered by the D60 system; future work should quantify irradiance and dose distribution. Additionally, real-world testing with clinical biofilms or organic loads beyond BSA should be pursued, as these factors can attenuate UV efficacy. Finally, extending inactivation studies to emergent enveloped viruses (e.g., SARS-CoV-2) under EN 14476 frameworks would broaden the system’s validation for pandemic preparedness [26].

## Conclusion

The present study provides compelling evidence for the high-level virucidal efficacy of the D60 UV-C disinfection system, demonstrating consistent >4.0 lg reductions across all EN 14476 indicator viruses—murine norovirus (MNV), poliovirus type 1, adenovirus type 5, and simian virus 777 (SV40)—within a 60 second disinfection cycle. These findings not only confirm the D60 system’s compliance with European biocidal regulatory standards (Regulation EU No. 528/2012) but also position it as a viable alternative to traditional chemical and thermal high-level disinfection (HLD) methods for semi-critical medical devices, particularly in high-throughput clinical disciplines such as otorhinolaryngology.

## Notes

### Authors’ ORCIDs

- Rudhart SA: https://orcid.org/0000-0003-3580-4255
- Eichhorn KWG: https://orcid.org/0000-0002-5840-4505
- Rausch M: https://orcid.org/0000-0003-1562-4337
- Maas A: https://orcid.org/0000-0001-8513-7572
- Knof M: https://orcid.org/0009-0001-9928-2057
- Engelhart S: https://orcid.org/0009-0008-8971-5892
- Mutters NT: https://orcid.org/0000-0002-0156-9595
- Strieth S: https://orcid.org/0000-0003-0177-1926

### Authors’ contribution

Stefan Alexander Rudhart and Klaus Wolfgang Georg Eichhorn contributed equally to this work and equally share the first authorship.

### Ethical approval 

The study was reviewed by the Ethics Committee of the University Hospital Bonn (reference number 2025-85-BO).

### Funding

None. 

### Competing interests

Stefan Alexander Rudhart reports equipment or supplies was provided by UV Smart Technologies B.V. Stefan Alexander Rudhart reports a relationship with UV Smart Technologies B.V. outside the submitted work that includes: consulting or advisory, speaking and lecture fees, and travel reimbursement. Maximilian Knof reports a relationship with UV Smart Technologies B.V. outside the submitted work that includes: consulting or advisory. Marvin Rausch reports a relationship with UV Smart Technologies B.V. outside the submitted work that includes: consulting or advisory. The other authors declare that they have no known competing financial interests or personal relationships that could have appeared to influence the work reported in this paper.
